# Effects of Mixed Isoenergetic Meals on Fat and Carbohydrate Metabolism during Exercise in Older Men

**DOI:** 10.1155/2011/172853

**Published:** 2011-05-24

**Authors:** Minoo Bassami, Donald P. M. MacLaren, Sajad Ahmadizad, Dominic Doran

**Affiliations:** ^1^Department of Sports and Exercise Physiology, Sports Science Research Centre, Ministry of Science, Research and Technology, 1587958711 Tehran, Iran; ^2^Research Institute for Sport & Exercise Sciences, Liverpool John Moores University, Liverpool L3 3AF, UK; ^3^Department of Sports and Exercise Physiology, Faculty of Sports Sciences, Shahid Beheshti University, Tehran, Iran

## Abstract

The present study was designed to investigate the effects of four different meals on fat and CHO metabolism during subsequent exercise in elderly males. Eight healthy males (age: 63.3 ± 5.2 years) reported to the physiology laboratory on four separate occasions, each of which was allocated for the performance of a 30-minute exercise on a cycle ergometer at 60% ${\dot{\text{V}}\text{O}}_{2\max\;}$ after having normal (N), high fat (HF), high carbohydrate high glycaemic index (HGI) and high carbohydrate low glycaemic index (LGI) meals. Fat oxidation during exercise after the meals (HF = 0.26 ± 0.04 g/min; N = 0.21 ± 0.04 g/min; HGI = 0.22 ± 0.03 g/min; LGI = 0.19 ± 0.03 g/min) was not significant (*P* > .05), and neither were the rates of carbohydrate oxidation (N = 1.79 ± 0.28, HF = 1.58 ± 0.22, HGI = 1.68 ± 0.22, and LGI = 1.77 ± 0.21 g/m). NEFA concentration increased after HF (*P* < .05) but decreased after HGI and LGI (*P* < .05). Glucose concentration decreased as a result of exercise after HF, and LGI (*P* < .05) whereas insulin concentration decreased significantly during exercise after N, HF, and HGI (*P* < .05). It can be concluded that, in elderly males, feeding isoenergetic meals containing different proportions of carbohydrate and fat do not significantly alter oxidation of fat and CHO during exercise in spite of changes in some circulating metabolites.

## 1. Introduction

A characteristic of ageing is an increase in adiposity and loss of muscle mass [1]. Since the increase in adiposity is related to general poor health factors such as an increase in type II diabetes and increased incidence of coronary heart disease, exercise to reduce adiposity is recommended for the ageing population. A further consequence of the ageing process is that elderly individuals have an impaired ability to oxidise fatty acids [2], particularly after a meal [3]. Since most individuals eat a meal prior to exercise in order to provide some form of sustenance, what should the meal contain if the exercise priority is to “burn fat”?

Generally, a high-fat, low-CHO meal increases fat oxidation during subsequent exercise [4–6], whereas the ingestion of CHO before exercise depresses the rate of fat oxidation due to hyperinsulinemia in the postprandial period [7]. Altering the type of CHO consumed has been shown to have an effect on the magnitude of hyperinsulinemia and depression of fat oxidation [8, 9] Postprandial increases in glucose and insulin concentration promote CHO oxidation, resulting in decreased fatty acid oxidation [10]. Wu et al. [9] found that the amount of fat oxidised was significantly higher during exercise commencing 3 h after consuming a low glycemic index (LGI) meal compared with a high glycemic index (HGI) meal. They also demonstrated that the HGI meal resulted in a greater glycemic and insulinemic response during the postprandial period compared with LGI meal. This is supported by Stevenson et al. [11] who investigated the metabolic responses to HGI and LGI mixed meals after 60-minute exercise at 70% ${\dot{\text{V}}\text{O}}_{2\max\;}$ and found that significant differences in hyperglycemia and hyperinsulinemia can be achieved repeatedly by changing the Glycemic Index (GI) of the CHO in a mixed meal. They observed that the amount of fat oxidised during the postprandial period following lunch was significantly higher in the LGI than the HGI trial. Thus, at least for several hours postprandial, both at rest and during exercise, fat utilisation is depressed after HGI compared with LGI meals.

The effects of different meals (high fat, HGI, and LGI) on fat and CHO metabolism at rest and during exercise in young subjects have been extensively studied, although this is not the case for elderly individuals. As mentioned previously, it is an important health benefit for the elderly to engage in some form of aerobic exercise for improvements in the cardiovascular system and to reduce body fat. Therefore, the present study was designed to investigate the effects of four different types of meals (normal, high-fat, HGI, and LGI) on fat and CHO metabolism during exercise in elderly male subjects.

## 2. Methods

### 2.1. Participants

Eight healthy males (Mean ± SD, age 63.3 ± 5.2 years, height 168 ± 0.05 cm, body mass 78.1 ± 14.0 kg, body fat 21 ± 5.3%, and $\dot{\text{V}}{\text{O}}_{2\;\max\;}$ 36.9 ± 10.4 ml·kg^−1^·min^−1^) gave informed written consent to participate in the study after gaining approval from the Human Ethics Committee of Liverpool John Moores University. Blood pressure (Dinamap Pro Series, GE Medical Systems, Florida) was determined prior to performing any exercise as a screening for hypertension.

### 2.2. Experimental Design

Participants reported to the laboratory on five separate occasions. In the first session they were familiarised with the laboratory environment and physiological testing equipment. Height, body mass, and percent of body fat using DXA were also determined during this session. After familiarisation, $\dot{\text{V}}{\text{O}}_{2\;\max\;}$ was determined on a cycle ergometer as described previously [4]. After initial physiological measurements, participants reported to the physiology laboratory on four separate occasions, each of which was allocated for the performance of a 30-minutes exercise on a cycle ergometer at 60% $\dot{\text{V}}{\text{O}}_{2\;\max\;}$ after having a high fat (HF), high carbohydrate HGI (HGI), high carbohydrate low LGI (LGI), and normal (N) meal. The rationale for 30-minutes exercise reflects a typical aerobic bout of exercise undertaken by such persons in a gym session and is recommended for health purposes. The four meals were given to subjects in a counterbalanced design and sessions were separated by at least 3 days. To avoid circadian variation, experiments were always performed at the same time of day (08:00 am) after an overnight fast. Participants completed a 2-day food diary on the day before their first test and were asked to repeat this diet before all subsequent trials. In addition, subjects were requested to refrain from drinking alcohol, nor to engage in any kind of strenuous exercise 24 hours before trials.

### 2.3. Exercise Protocol

Participants reported to the physiology laboratory after an overnight fast and remained seated for 20 minutes. After this rest period, blood pressure was checked and a blood sample (10 mL) was taken. They then consumed one of the meals which were provided in a random, counterbalanced order within 20–30 minutes. At 3 h 20 min after the meal, subjects started the exercise protocol that included a 30-minutes cycling at 60% $\dot{\text{V}}{\text{O}}_{2\;\max\;}$. Two more venous blood samples were taken, immediately before exercise (3 hours after the meal), and immediately after exercise in each session. Oxygen consumption (Vo_2_), carbon dioxide output (Vco_2_), and respiratory exchange ratio (RER) were measured breath by breath throughout the exercise. Rates of fat and carbohydrate oxidation were calculated using the equations of Frayn [12].

### 2.4. Dietary Analysis

Participants were provided with one of the four following isoenergetic test meals: (1) and (2) HGI and LGI: 65% carbohydrate, 20% fat, and 15% protein, (3) HF: 65% fat, 20% carbohydrate and 15% protein, or (4) N: 50% carbohydrate, 35% fat, and 15% protein. The glycaemic index values for HGI and LGI were 74.32 and 29.26, respectively.

### 2.5. Blood Sampling and Analyses

Before the meal, immediately before exercise and immediately after exercise venous blood samples were drawn in a seated position in each session. Two microhaematocrit tubes (L. I. P. Shipley, England) and two *β*-haemoglobin microcuvettes (Hemocue AB, Ängleholm, Sweden) were filled with whole blood for determination of haematocrit and haemoglobin, respectively. Changes in plasma volume were subsequently calculated using the equation of Dill and Costill [13].

Plasma was obtained by collecting the blood sample into tubes that had been pretreated with an anti-coagulant (lithium heparin). These samples were mixed and immediately centrifuged at 4°C for 15 minutes at 1900 g. After centrifugation, plasma was separated and stored at −70°C for the subsequent analysis of glucose, glycerol, nonesterified fatty acids (NEFAs), and B-hydroxybutyrate (3-OHB). Serum was obtained by collecting blood samples into serum separation tubes. The blood was stored at room temperature for 30 minutes before centrifuged at 20°C for 15 minutes at 2000 g. Serum was stored at −70°C for subsequent analysis of Insulin.

NEFA, Glucose, Glycerol, and 3-OHB were analysed using appropriate kits on ILab 300 analyser (IL Instrumentation laboratories, Warrington, UK). Insulin was assayed by ELISA (DRG instruments Gmbh, Germany) using a fully automated system (Triturus, Grifols, Cambridge, UK).

Insulin resistance (HOMA2-IR) and *β*-cell function (HOMA2 %B) in fasting state were determined using a homeostasis model assessment (HOMA-IR) and were calculated from fasting insulin and fasting glucose [14].

### 2.6. Statistical Analysis

All statistical analyses were performed using the software statistical package SPSS version 12 (Chicago, USA). One-way ANOVA was employed to evaluate differences in the resting mean values of all the variables measured over the four testing occasions. In addition, fat and CHO oxidations values during exercise for four trials were compared using one-way ANOVA. A two-way ANOVA (4 × 3) with repeated measures across meals (4 levels) and conditions (3 levels) was employed to examine the differences in mean values for blood parameters. When ANOVA indicated the presence of a significant difference, *post hoc* comparisons using the Bonferroni method were applied to determine pairwise differences. Values in the text are presented as mean (±SE) unless otherwise stated. The level of significance in all statistical analyses was set at *P* < .05.

## 3. Results

### 3.1. Substrate Oxidation

No significant main effect of the meals was observed for rates of fat oxidation (*F*_3,21_ = 1.8; *P* = .177), although fat oxidation was demonstrably, but nonsignificantly, higher after HF (0.26 ± 0.04 g/min) than N (0.21 ± 0.04 g/min), HGI (0.22 ± 0.03 g/min), and LGI (0.19 ± 0.03 g/min). The rates of carbohydrate oxidation during exercise were 1.79 ± 0.28, 1.58 ± 0.22, and 1.68 ± 0.22, 1.77 ± 0.21 g/min for N, HF, HGI, and LGI, respectively. Statistical analysis revealed no significant effect of meal on the rate of carbohydrate oxidation during exercise.

### 3.2. Blood Parameters

**Figure 1:**
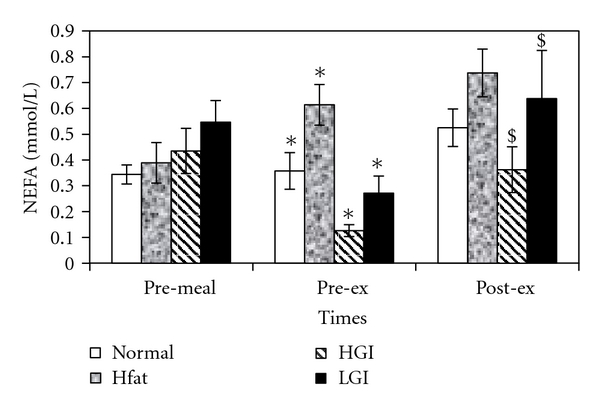
Mean (±SE) plasma NEFA concentrations at pre-meal, pre-exercise, and post-exercise for four trials. ∗ indicates significant with resting values and $ indicates significant difference with postprandial values.

Statistical analysis showed a significant main effect of the meal on NEFA concentration (*F*_3,21_ = 39.2; *P* = .001). Pairwise comparisons revealed a significant difference between NEFA responses to N and HF (*P* = .02) as well as between HGI and LGI meals (*P* = .003). Pre-exercise NEFA concentration increased significantly following HF (from 0.39 ± 0.08 to 0.61 ± 0.08 mmol/L) and decreased significantly after eating HGI (from 0.44 ± 0.09 to 0.13 ± 0.02 mmol/L) and LGI (from 0.55 ± 0.08 to 0.27 ± 0.07 mmol/L). However, NEFA concentration increased significantly in response to exercise only after HGI (from 0.12 ± 0.02 to 0.36 ± 0.09 mmol/L) and LGI (from 0.27 ± 0.06 to 0.64 ± 0.18 mmol/L).  Figure 1 highlights data from NEFA.

**Figure 2:**
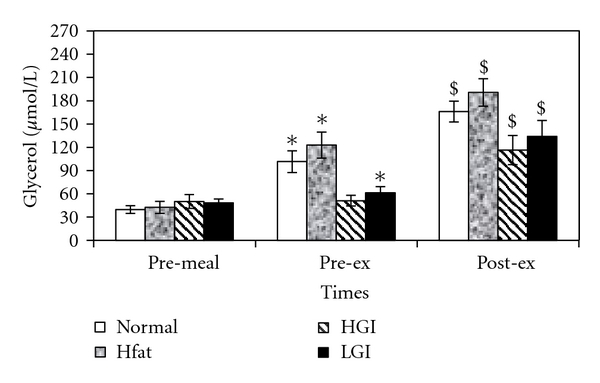
Mean (±SE) plasma glycerol concentrations at pre-meal, pre-exercise and, post-exercise for four trials. ∗ indicates significant with resting values and $ indicates significant difference with postprandial values.

A significant effect of meal was found for glycerol concentration (*F*_3,21_ = 9.7; *P* = .001). Pairwise comparisons revealed a significant difference between HF and HGI (*P* = .01). Resting glycerol values were increased significantly after all meals except for HGI (Figure 2). Moreover, glycerol concentration increased significantly during exercise after all types of meals (Figure 2).

**Figure 3:**
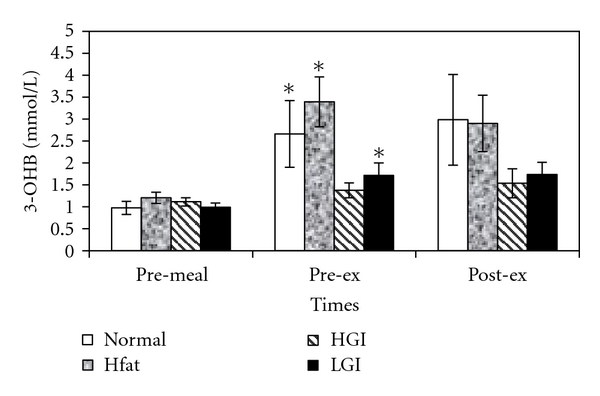
Mean (±SE) plasma 3-OHB concentrations at pre-meal, pre-exercise and, post-exercise for four trials. ∗ indicates significant with resting values.

The statistical analysis revealed a significant effect of the meal on 3-OHB (*F*_3,21_ = 3.6; *P* = .03). However, pairwise comparisons did not show any significant difference among the four meals. Resting 3-OHB concentration increased significantly after all meals except for HGI (Figure 3). 

**Figure 4:**
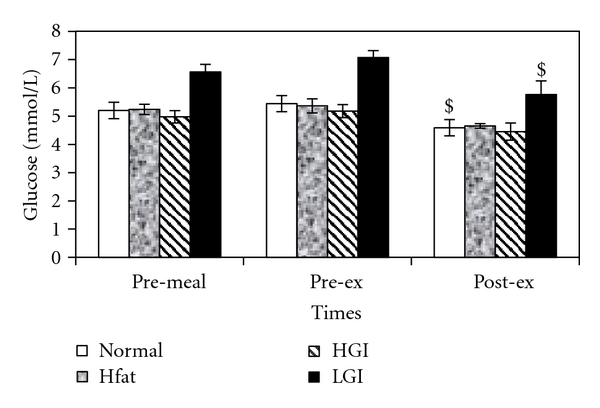
Mean (±SE) plasma glucose concentrations at pre-meal, pre-exercise and, post-exercise for four trials. $ indicates significant difference with postprandial values.

A significant effect of meals on glucose concentration was found (*F*_3,21_ = 39.2; *P* = .001). Postprandial glucose concentration increased following all meals, though the changes were not statistically significant (Figure 4). However, glucose concentration decreased significantly (*P* < .05) from 5.36 ± 0.25 to 4.65 ± 0.08 mmol/L and from 7.18 ± 0.25 to 5.76 ± 0.48 mmol/L during 30 minutes of exercise after HF and LGI, respectively.

**Figure 5:**
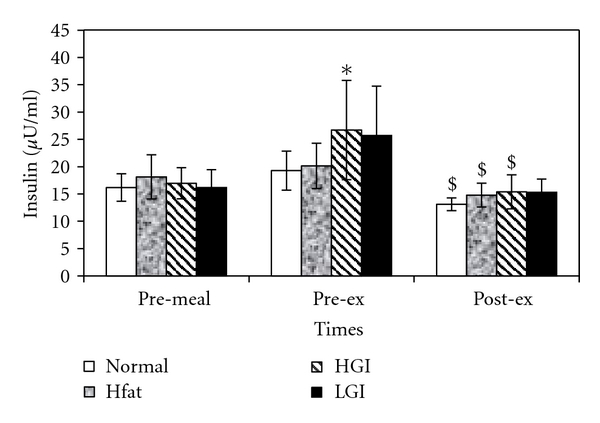
Mean (±SE) plasma insulin concentrations at pre-meal, pre-exercise and, post-exercise for four trials. ∗ indicates significant with resting values and $ indicates significant difference with postprandial values.

Although ANOVA did not show a main significant effect of either meal or time on insulin concentration, the pre-exercise insulin concentration (18.9 ± 1.7 *μ*U/mL) in HGI trial was significantly (*P* < .05) higher than resting values (15.2 ± 1.1 *μ*U/mL). In addition, insulin concentration decreased significantly during exercise after N, HF and HGI (Figure 5). The mean (±SE) resting value of HOMA2-IR (insulin resistance) for all subjects was 0.76 ± 0.06 and that for HOMA2-*β* (*β*-cell function) was 78.5 ± 69. These resting values are indicators of normal insulin resistance and *β*-cell function in our elderly subjects.

## 4. Discussion

The present study is the first study designed to investigate the effects of pre-exercise mixed meals on fat and carbohydrate metabolism during exercise in elderly individuals, and its principle finding was that in spite of some changes in fat metabolites, the composition of the meal did not result in differences in CHO and fat oxidation. These results are in contrast to the data reported by previous studies in young subjects that observed a rise in fat oxidation after HF due to increases in fatty acid (FA) availability and mobilization [4, 6, 15, 16]. Moreover, a depression in the rate of fat oxidation following CHO ingestion, attributed to hyperinsulinaemia, has also been observed in the postprandial period [7–9]. 

Availability and utilisation of plasma FA decreases after a CHO meal partly because the CHO-induced rise in insulin inhibits the mobilisation and availability of circulating FA, which reduces fat oxidation possibly by inhibiting the rate of long-chain fatty acids entry into the mitochondria for *β*-oxidation [17, 18]. Despite CHO-induced hyperinsulinemia and suppression of FA after CHO ingestion, our results show that the CHO oxidation was resistant to alteration during exercise in elderly males. Several mechanisms, including impaired insulin-stimulated glucose uptake, may have contributed to these results. Having said that, it should be noted that the HOMA scores indicate normal insulin resistance and *β*-cell function for these elderly participants.

The increased release of FAs in older individuals, in excess of the energy needs and/or oxidative capacity of respiring tissues, increases the amount of non-oxidised FAs. Excess non-oxidised FAs with age may have several adverse metabolic effects including increased glucose production [19] and impaired insulin-stimulated glucose uptake [20]. FAs exert their effects through inhibition of PDH with subsequent increased intracellular concentration of glucose-6-phosphate and inhibition of hexokinase, which decreases glucose uptake [21]. Another factor that may contribute to impaired glucose uptake with ageing is inhibition of glucose transport either due to less availability of the GLUT-4 transporters or, to the signalling processes for GLUT-4 vesicle translocation to the plasma membrane [22]. These considerations need further exploration.

Although after the HF meal, NEFA, and glycerol concentrations were higher than for the other meals, the fat oxidation rates during exercise were not different. This contradicts the findings in young participants [4, 15, 16]. Evaluation of skeletal muscle samples has revealed that maximal mitochondrial oxidative enzyme activity is lower in older than in young subjects because of both decreased mitochondrial volume density [23] and mitochondrial function [24]. Lower activity of enzymes such as AMPK, cAMP, and protein kinase C in elderly individuals results in activation of ACC (acetyl-CoA carboxylase). Activation of ACC leads to an increase in concentration of malonyl-CoA which has an inhibitory effect on CPT-I, thereby inhibiting the entry of long chain fatty acids into mitochondria and resulting in lower FA oxidation [25]. We did not examine activities of such enzymes and so can only speculate.

Lack of changes in fat oxidation after HF was found in spite of increased lipolysis. It has been demonstrated that relative to the energy needs of the body, FA release is not impaired in the elderly [26]. In fact, FAs are released in excess of energy needs in older individuals when compared to younger controls. Thus, when considered relative to the energy demands of the body or the metabolically active tissue mass, ageing is not associated with impaired FA release which supports our findings for lipolysis. 

A higher rate of fat oxidation during submaximal exercise after ingesting LGI foods has been reported in young healthy males when compared to HGI [9, 11]. In terms of the effect of GI, the results of the present study were somewhat unexpected since the calculated amount of fat oxidation during 30 minutes of cycling commencing 3 hour after consuming HGI and LGI meals was not significantly different. One possible explanation for this discrepancy might be the impaired glucose uptake associated with ageing as previously discussed.

In the present study, postprandial NEFA concentration was increased after HF, which is in agreement with previous studies on young participants [6, 9]. The increase in plasma NEFA concentration which occurred might be a result of TAG hydrolysis by endothelial lipoprotein lipase (LPL). Both HGI and LGI resulted in suppression of NEFA concentrations 3 hours after the meal consumption. However, NEFA concentrations at the end of 30-minutes cycling in both HGI and LGI trials were raised to pre-meal levels. Higher post-exercise NEFA is typical response to exercise-induced decrease in insulin and increase in catecholamines. 

Postprandial glycerol concentration increased significantly after all meals except for HGI. The lack of a significant increase in glycerol after HGI might be due to the enhanced secretion of insulin following the HGI meal which would activate the enzyme LPL in adipose tissue [27]. The insulin activation of LPL serves to increase TAG uptake and storage after a single meal which eventually results in lower glycerol concentration. Studies in young subjects during low and moderate intensity exercise have demonstrated that increased blood glucose availability suppresses fat utilisation by inhibition of both fat mobilisation and fat oxidation within muscle [7, 18]. Postprandial glycerol concentration was significantly higher after HF than HGI and LGI in elderly subjects which reflects the lack of insulin response to HF in comparison to the other meals. These findings are in agreement with those of Whitley et al. [6] and Murphy et al. [28] who reported increases in glycerol after HF in young participants.

The present study demonstrated that in elderly individuals feeding isoenergetic meals containing different proportions of carbohydrate and fat alters the metabolic variables at rest and during subsequent exercise. Energy regulation during 30 min of cycling in elderly individuals following isoenergetic meals is associated with a relative increase in fat oxidation and a decrease in carbohydrate oxidation following HF and a corresponding increase in CHO oxidation and a decrease following high carbohydrate (low fat) meals. Therefore, based on these finding, it could be concluded that fat and carbohydrate metabolism in elderly individuals in response to different meals at rest and during subsequent exercise are to some extent different from those of young individuals and further studies are warranted to investigate the mechanism/s responsible especially in relation to insulin action and sensitivity. What we can state is that, on balance, eating any type of meal 3 hours prior to a 30-minutes bout of exercise is unlikely to significantly impact on so-called “fat burning” in healthy elderly males.
